# Dynamic identification of candidate genes associated with higher tocopherol biosynthesis in *Brassica napus* seeds

**DOI:** 10.3389/fpls.2025.1613360

**Published:** 2025-06-05

**Authors:** Yunyun Zhang, Ping Qin, Yajun Liu, Yingnan Liu, Wenjing Li, Chunjuan Luo, Peng Chen, Chunyu Zhang

**Affiliations:** ^1^ College of Plant Science and Technology, Huazhong Agricultural University, Wuhan, Hubei, China; ^2^ Yunnan Key Laboratory of Genetic Improvement of Herbal Oil Crops, Industrial Crops Research Institute, Yunnan Academy of Agricultural Sciences, Kunming, China; ^3^ Hunan Institute of Engineering, Xiangtan, Hunan, China; ^4^ Lincang Agricultural Technology Extension Center, Lincang, Yunnan, China; ^5^ Wuhan Agricultural Technology Extension Center, Wuhan, China

**Keywords:** *Brassica napus*, vitamin E, tocopherol biosynthesis, transcriptome, BSA-seq, candidate genes

## Abstract

Vitamin E is a crucial fat-soluble antioxidant playing vital roles in human health as well as the growth and development of plants and animals. *Brassica napus* L. (rapeseed) is recognized as the world’s second most important oilseed crop, serving as a primary source of vegetable oil and vitamin E. However, the regulatory network governing vitamin E biosynthesis during rapeseed seed development remains poorly understood. In this study, transcriptome analyses were conducted using two pairs of rapeseed germplasms with high-VE (YH) or low-VE (YL) contents across entire seed developmental stages (15–50 DAF, days after flowering). The relationship between chlorophyll catabolism and vitamin E accumulation was systematically investigated, and candidate genes associated with seed VE biosynthesis were identified. Key findings include greater vitamin E accumulation observed in the high-VE line, primarily attributed to sustained VE biosynthesis during late seed development (45–50 DAF). Through transcriptome and weighted gene co-expression network analysis (WGCNA) during late seed development (35–50 DAF), four key regulatory modules were revealed, highlighting seven hub genes involved in chlorophyll catabolism and vitamin E biosynthesis. Additionally, two candidate loci on chromosomes A03 and C08 were identified via bulked segregant analysis sequencing (BSA-seq), along with five candidate genes (e.g., *BnA03g0107720*) proposed as critical regulators for *B. napus* seed vitamin E biosynthesis. These results contribute to an advanced understanding of the regulatory mechanisms underlying seed VE biosynthesis in oilseed crops and provide valuable genetic resources for enhancing rapeseed nutritional quality through vitamin E biofortification.

## 1 Introduction

As a globally significant oilseed crop, rapeseed (*Brassica napus* L.) serves not only as a vital source of dietary fats, oils, and proteins for humans but also as an indispensable raw material in industrial production. Its seeds are rich in tocopherols (vitamin E), a compound with potent antioxidant and free radical-scavenging properties. Since vitamin E, an essential nutrient, cannot be synthesized endogenously by humans or animals, exogenous intake is imperative (Dror and Allen, 2011; Liu et al., 2024). Nutritional studies confirm a widespread chronic deficiency of vitamin E across global populations (Kim and Cho, 2015). Adequate vitamin E intake not only prevents neurodegenerative diseases but also mitigates chronic conditions such as atherosclerosis, cataracts, and malignancies (Cerqua et al., 2022; Jiang, 2022). In animal husbandry, dietary vitamin E supplementation enhances the oxidative stability of meat products and extends shelf life (Bellés et al., 2018; Leal et al., 2018; Alsamadany and Ahmed, 2022). From a plant physiological perspective, vitamin E plays a critical role in improving abiotic stress tolerance by scavenging reactive oxygen species (Havaux et al., 2005), maintaining membrane integrity (Niu et al., 2022), and participating in signal transduction (Fleta-Soriano and Munné-Bosch, 2017; Fang et al., 2019; Ma et al., 2020; Liu et al., 2024). Additionally, vitamin E regulates seed storage stability, seed vigor maintenance, and oil quality (Hunter and Cahoon, 2007; Zhang et al., 2007; Kiyose, 2021; Alsamadany and Ahmed, 2022).

Since the cloning of the first plant tocopherol biosynthesis gene *HPPD* in *Arabidopsis thaliana* (Norris et al., 1998), researchers have systematically elucidated core metabolic pathways of tocopherol biosynthesis in maize (*Zea mays*), soybean (*Glycine max*), and sunflower (*Helianthus annuus*) using multi-omics approaches (Cheng et al., 2003; DellaPenna, 2005; Vom Dorp et al., 2015; Wang et al., 2018; Ghosh et al., 2022). Notably, recent studies have revealed metabolic coupling between tocopherol biosynthesis and chlorophyll cycling, with key chlorophyll metabolic enzymes—including chlorophyll synthase (CHL), chlorophyll dephytylase 1 (CLD1), phytyl kinase (VTE5), and phytyl phosphate kinase (VTE6)—directly regulating tocopherol biosynthesis (Valdez-Agramón et al., 2022; Muñoz et al., 2024; Romer et al., 2024). Genome-wide association studies (GWAS) have identified a novel esterase gene (*VTE7*) localized to the chloroplast envelope, significantly influencing vitamin E synthesis (Albert et al., 2022). Recently, *BnaC02.VTE4* was identified as the causal factor for divergent vitamin E and glucosinolate contents in rapeseed through GWAS. Moreover integrated transcriptomic and metabolomic analyses further clarified substrate competition between vitamin E and glucosinolate biosynthesis pathways (Wang et al., 2025) in *B. napus*. In our prior work in Arabidopsis, we identified a hydrolytic enzyme, pheophytin pheophorbide hydrolase (PPH), and discussed the critical role of chlorophyll synthase in vitamin E biosynthesis (Zhang et al., 2013, 2015). Our recent work showed *CHLSYN* suppression and homogentisic acid (HGA) synthesis enhancement successfully elevated seed vitamin E content in Arabidopsis (Qin et al., 2024). Although chlorophyll metabolites are recognized as phytol donors for tocopherols, the mechanisms underlying phytol release for tocopherol synthesis remain elusive.

System biology approaches integrating multi-omics approach have advanced the molecular dissection of quality traits in crops. Studies in maize (*Zea mays*), soybean (*Glycine max*), and citrus (*Citrus* spp.) have delineated dynamic regulatory networks of tocopherol biosynthesis and their interactions with photosynthetic metabolism (Li et al., 2019, 2021; Peng et al., 2023). Intriguingly, interspecific variations exist in chlorophyll-tocopherol coordination: during pomelo (*Citrus grandis*) fruit development, chlorophyll exhibited a strong positive correlation with α-tocopherol (r > 0.9) but a negative correlation with γ-tocotrienol (r< -0.9), while in pummelo (*Citrus maxima*), only α-tocotrienol was found to be negatively correlated with chlorophyll (Zhao et al., 2021). Studies in maize confirmed strong positive correlations between chlorophyll a/b, chlorophyllide a, and total tocopherol levels (Diepenbrock et al., 2017). While in soybean a more complex regulation was found, with α- and γ-tocopherols positively correlated with chlorophyll contents (r = 0.372, p< 0.05), and δ-tocopherol negatively correlated (r = -0.221, p< 0.05) (Vinutha et al., 2017).

As the second-largest global oilseed crop, the seeds of *Brassica napus* L. constitute a key dietary source of vitamin E. However, the key players in the regulatory network mediating chlorophyll degradation and tocopherol synthesis in oilseed crops remains unresolved, hindering the simultaneous improvement of oil quality (reduced chlorophyll residues) and nutritional value (enhanced tocopherol content). In this study, we integrates BSA-Seq and RNA-Seq to systematically investigate dynamic changes on chlorophyll and tocopherol contents during whole seed developmental stages in *Brassica napus*. We delineated spatiotemporal coupling between chlorophyll synthesis/degradation and tocopherol biosynthesis pathways, characterize key regulatory modules and candidate genes governing metabolic flux partitioning between high- and low-vitamin E accessions. These findings provide novel insights into the regulatory network of vitamin E biosynthesis in oilseed crops, and set pavement for future breeding work to improve seed vitamin E levels in oilseed rape.

## 2 Materials and methods

### 2.1 Plant materials

In the initial phase of this study, four rapeseed varieties with stable differences in total vitamin E content in their seeds were identified: YH01, YH07, YL02, and YL04. Among the varieties, YH01 and YH07 were classified as high vitamin E content lines (YH), with average seed vitamin E contents over two years recorded at 527.85 µg/g and 523.20 µg/g, respectively. Conversely, YL02 and YL04 were categorized as low vitamin E content lines (YL), with average seed vitamin E contents of 244.49 µg/g and 383.73 µg/g, respectively, over the same period. These varieties were cultivated in the experimental rapeseed field at Huazhong Agricultural University in Wuhan (114° E, 30° N) during the 2020/2021 and 2021/2022 growing seasons. Each variety was planted in 20 rows, with three replications, maintaining 8–10 plants per row, and spaced 21 cm apart. The row spacing was set at 30 cm, and standard water and fertilizer management practices were followed throughout the entire growth period.

Developing seed samples were collected every five days from 15 to 70 DAF (days after flowering) for each of the four varieties, with three biological replicates at each time points, resulting in a total of 144 samples (**Supplementary Table S1**). The samples were rapidly frozen in liquid nitrogen and stored at -80°C for subsequent analysis.

Furthermore, we utilized the high-vitamin E (VE) accession YH07 and low-VE accession YL02 as parental materials to set up a cross, microspore culture was performed on F_1_ progeny to generate an DH (doubled haploid) population. Following cultivation, mature seeds from the DH population were harvested for statistical analysis of seed vitamin E content. Fresh leaf tissues from the DH population were collected and flash-frozen in liquid nitrogen and stored at -80°C freezer for DNA extraction.

### 2.2 High performance liquid chromatography analysis of tocopherol and composition

An HPLC with the fluorescence detector was used for quantification of seed tocopherol contents, using 5, 7-dimethyltocol (Matreya, www.matreya.com) as internal standard (Yang et al., 2011). 50 mg low-temperature lyophilized sample, 1.5 ml of methanol/dichloromethane (9:1 v/v) and 60μl internal standard were added to a 2 ml centrifuge tube, The sample were then ground with steel balls, then incubated at room temperature for 3 hrs. Tocopherol compounds were separated by an Agilent Eclipse XDB-C18 reversed-phase column (4.6×150mm, 5μm particle size, www.agilent.com), with isocratic conditions of methanol/water (95:5 v/v) at flow rate of 1.5 ml/min. The abundance of each type of tocopherol was monitored by excitation at 292 nm and emission at 330 nm as previously described (Yang et al., 2011).

### 2.3 Extraction and determination of chlorophyll and composition

The method for spectrophotometric determination of chlorophyll was conducted as described (Arnon, 1949). For chlorophyll extraction, the freeze-dried seed samples were weighed and homogenized in liquid nitrogen, and subsequently extracted with 3 volumes of 80% (v/v) acetone containing 1 mM KOH. After centrifugation at 12000 rpm for 2 min, supernatants were collected and used for spectrophotometric measurements.

The contents of chlorophyll a, chlorophyll b and total chlorophyll were calculated according to the following equations.


$$\text{ Ca }\left(\text{Chlorophyll a},\text{ mg}/\text{L}\right)=12.7A_{663}-2.69A_{645}$$

$$\text{Cb }\left(\text{Chlorophyll b},\text{ mg}/\text{L}\right)=22.9A_{645}-4.68A_{663}$$

CT (Total chlorophyll, mg/L)=Ca+Cb

$$\text{Chlorophyllcontent}\left(\text{mg}/\text{g}\right)=\frac{\text{C }\left(\text{mg}/\text{L}\right)\;\times \text{acetone volume }\left(\text{L}\right)}{\text{sample weight}\left(\text{g}\right)}$$

A=Absorbance

Data were expressed as milligrams per gram of sample dry weight (mg/g DW), and standard deviation was calculated using data from three biological replicates.

### 2.4 Sequencing and transcriptomic data analysis

Seeds at 15, 25, 35, 45and 50 DAF were collected from the four rapeseed accessions (YH01,YH07,YL02 and YL04), respectively. Three biological replicates were taken at each time point. Total RNA was prepared with TRIzol Reagent Kit (Ambion) according to the manufacture’s protocol. RNA samples were treated with RNase-free DNase I (Tiangen, Beijing, China). RNA quality and concentration were monitored using Agilent 2100 Bioanalyzer (Agilent Technologies, Inc., Santa Clara, CA, USA) and Nanodrop 2000 (Thermo Scientific, Wilmington, MA, USA), respectively. RNA samples were sent to Shanghai Personal Biotechnology Co., Ltd. for library construction and sequencing on the NovaSeq 6000 platform (NovaSeq 6000; Illumina, San Diego, CA, USA). Reads were mapped to the ‘ZS11’ reference genome (https://yanglab.hzau.edu.cn/BnIR/germplasm_info?id=ZS11.v2) using HISAT2 (Kim et al., 2015), and the reads count data were analyzed using DESeq2 (Love et al., 2014). Gene Ontology (GO) enrichment was performed using TBtools (Chen et al., 2020).

### 2.5 Weighted gene co-expression network analysis and visualization

We conducted Weighted Gene Co-expression Network Analysis (WGCNA) and calculated the module feature values and the correlation between chlorophyll and tocopherol content to screen key genes and construct a co-expression network. We also visualized the WGCNA network of trait specific modules using Cytoscape 3.9.0.

### 2.6 Quantitative real-time PCR

qRT-PCR primers were designed using Primer3 (https://bioinfo.ut.ee/primer3-0.4.0/), the primer sequences were listed in **Supplementary Table 2**. cDNA was synthesized from the same set of samples used for RNA-Seq using EasyScript^®^ One-Step gDNA Removal and cDNA Synthesis SuperMix (TRANS). First-strand cDNA was synthesized from 1 µg total RNA using Ominiscript Reverse Transcriptase (Qiagen), and qRT-PCR was conducted using the Bio-Rad CFX96 real-time system (Bio-Rad Laboratories, Hercules, CA, USA). Gene relative expression was calculated using the 2^-ΔΔCt^ method (Livak and Schmittgen, 2001) with *BnaACTIN* used as reference gene.

### 2.7 DNA library construction and BSA-Seq

High vitamin E content material YH07 and low vitamin E content material YL02 were selected for crossing, and a DH population was generated through microspore culture on F1 plants during flowering. Seed vitamin E levels in the DH population were statistically analyzed, two extreme groups were selected with 20 lines for each group with high- or low- vitamin E contents. Whole-genome resequencing was then performed on these bulk-DNA samples using the Illumina HiSeq2000 platform (GENOSEQ, Wuhan, China) at a sequencing depth of 30×.

### 2.8 Statistical analyses

For seed total vitamin E measurement, plants at harvesting stage or at different developmental stages were collected in three biological replicates. Total tocopherol contents were measured and standard deviation (SD) was calculated using data from three biological replicates. Statistical significance was determined using student t-test; *, ** and *** represent p< 0.05, p< 0.01 or p< 0.001, respectively.

## 3 Results

### 3.1 Dynamic analysis of tocopherols and chlorophyll during seeds development between YH and YL

**Figure 1:**
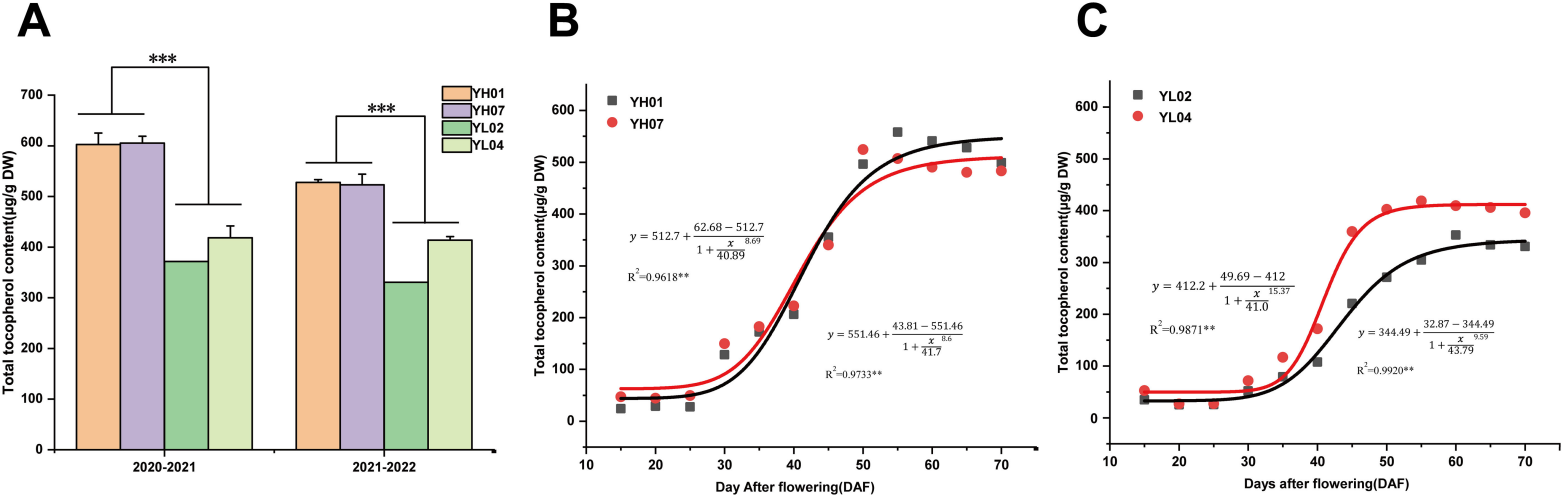
The content of tocopherols in high and low tocopherol rapeseed seeds. **(a)** Seed tocopherol content of high and low materials in Wuhan for two years. **(b)** Accumulation of total tocopherols and logistic equation fitting during the development of YH rapeseed seed. **(c)** Accumulation of total tocopherols and logistic equation fitting during the development of YL rapeseed seed. Asterisks on the lines indicate significant differences between the mean samples by Student’s t-test (***P<0.001).

In order to identify the optimal developmental stages for tocopherol accumulation in *Brassica napus* seeds, two pairs of *B. napus* accessions (YH07 and YH01, YL02 and YL04) with high or low seed tocopherol contents were cultivated in Wuhan over two consecutive years. Compared to YL materials, the YH materials exhibited average increases of 52.8% and 41.2% in seed tocopherol contents (**Figure 1a**). We then systematically quantified the dynamic changes in total tocopherol content in seeds from all four accessions from 15–70 DAF (days after flowering) with 5-day intervals. Total tocopherol content increased progressively during seed development, reaching a plateau at 50–60 DAF (**Figures 1b, c**). Among different types of tocopherols, γ-tocopherol predominated, followed by α-tocopherol, with δ-tocotrienol being the least abundant (**Supplementary Figure A–D**). Logistic modeling of total tocopherol accumulation (**Figures 1b, c**) revealed a sigmoidal curve, enabling division of the filling process into three phases: gradual increase stage (15–25 DAF), rapid increase stage (25–45 DAF), and stable increase stage (45–70 DAF). All four accessions initiated tocopherol synthesis during 15–25 DAF, with synthesis rates accelerating markedly post-25 DAF. The most pronounced difference between accessions occurred at 25–45 DAF, with seed color transition at 50 DAF.

Between 15–25 DAF, tocopherol content showed minimal variation between high- and low-VE accessions. At 35 DAF, YH accessions exhibited accelerated VE accumulation, reaching 172.4 μg/g dry weight (DW) and 182.4 μg/g DW, whereas YL accessions showed 79.0 μg/g DW and 116.9 μg/g DW, respectively. By 45 DAF, YH accessions achieved 355.3 μg/g DW and 340.2 μg/g DW, compared to 220.4 μg/g DW and 359.8 μg/g DW in YL accessions. At seed maturity (50 DAF), YH accessions accumulated 496.4 μg/g DW and 524.5 μg/g DW, significantly surpassing YL accessions (271.4 μg/g DW and 402.3 μg/g DW) (**Supplementary Figure S1**). These results demonstrated more pronounced tocopherol biosynthesis in YH accessions during seed development.

Chlorophyll dynamics across developmental stages are shown in **Supplementary Figure 2**. Both YH and YL accessions displayed synchronized chlorophyll accumulation patterns: rapid synthesis during early seed development peaking at 30 DAF, followed by progressive degradation until near-zero levels at maturity. Chlorophyll a consistently exceeded chlorophyll b throughout seed development, with both isoforms mirroring total chlorophyll contents (**Supplementary Figure S2A-D**). No significant difference in chlorophyll content were observed between accessions. All materials exhibited peak chlorophyll levels at 30 DAF (YH: 1.23 mg/g DW and 0.93 mg/g DW; YL: 0.83 mg/g DW and 0.98 mg/g DW), chlorophyll content declined sharply after 35 DAF, reaching 0.21 mg/g DW and 0.03 mg/g DW in YH compared to 0.10 mg/g DW and 0.06 mg/g DW in YL at 50 DAF. Therefore, chlorophyll contents change with two distinct phases: rapid synthesis from 15–35 DAF and degradation from 35–50 DAF. Notably, both chlorophyll a and b exhibited coordinated decline in YH and YL accessions during late seed development despite difference on tocopherol accumulation.

### 3.2 Dynamic analysis of chlorophyll and tocopherol accumulation during seed development

**Figure 2:**
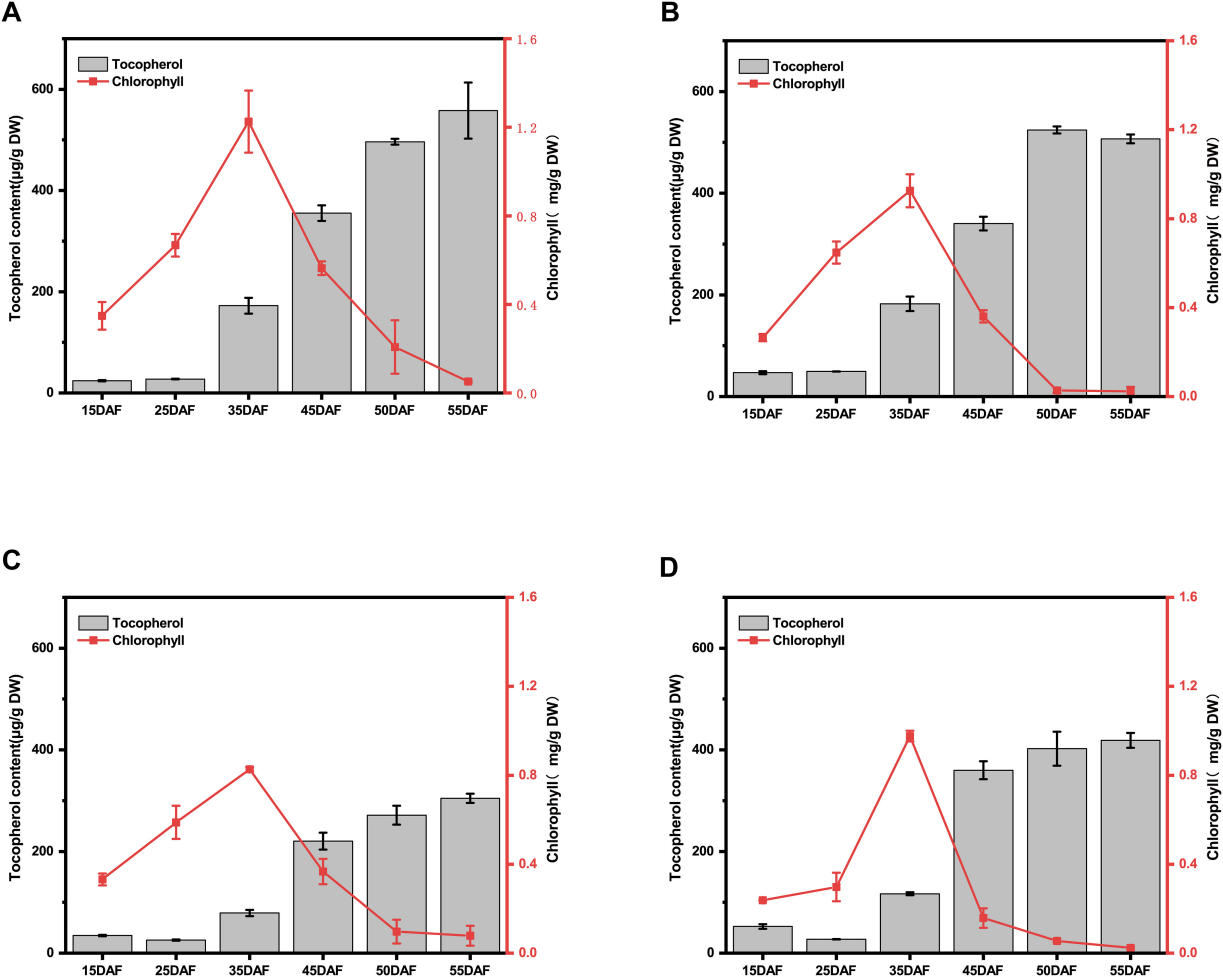
Analysis of the relationship between chlorophyll and tocopherols during the critical period of seed development. **(A)** Analysis of the relationship between chlorophyll and tocopherol during seed development in YH01. **(B)** Analysis of the relationship between chlorophyll and tocopherol during seed development in YH07. **(C)** Analysis of the relationship between chlorophyll and tocopherol during seed development in YL02. **(D)** Analysis of the relationship between chlorophyll and tocopherol during seed development in YL04.The left ordinates represents the total tocopherol content of the seeds, and right ordinates represents the total chlorophyll content of the seeds. The results of each material are based on three biological and three technical replicates. The error bars indicate standard errors.

The temporal accumulation patterns of chlorophyll and tocopherol (vitamin E) during *Brassica napus* seed development is critical for rapeseed nutritional quality improvement. We demonstrated that both chlorophyll and tocopherol exhibited pronounced synthesis during early seed development (15–35 DAF), while chlorophyll content declined rapidly from 35–50 DAF with tocopherol contents persisted at high levels in both high- and low-VE materials (**Figures 2A–D**).

Specifically, in the YH01 genotype, tocopherol content increased from 15 DAF (38.19 ± 1.17 μg/g DW) to 35 DAF (172.43 ± 15.69 μg/g DW), representing a 351.5% increment, with a subsequent 39.7% increase during 45–50 DAF. Chlorophyll content exhibited a 3.52-fold rise from 15 DAF (0.35 ± 0.06 mg/g DW) to 35 DAF (1.23 ± 0.24 mg/g DW), followed by rapid degradation (**Figure 2A**). For YH07, tocopherol accumulation surged by 288.6% (15 DAF: 46.94 ± 2.98 μg/g DW → 35 DAF: 182.41 ± 14.22 μg/g DW) and 54.2% (45–50 DAF), while chlorophyll content increased 3.49-fold (15 DAF: 0.26 ± 0.02 mg/g DW → 35 DAF: 0.93 ± 0.07 mg/g DW) before catabolic decline (**Figure 2B**).

In YL02, tocopherol content rose 128.1% between 15 DAF (34.63 ± 1.56 μg/g DW) and 35 DAF (78.99 ± 6.05 μg/g DW), with a 23.1% late-stage increment. Chlorophyll accumulation peaked at 2.49-fold (15 DAF: 0.33 ± 0.03 mg/g DW → 35 DAF: 0.83 ± 0.01 mg/g DW) prior to degradation (**Figure 2C**). YL04 demonstrated a 122.7% tocopherol increase (15 DAF: 52.48 ± 4.59 μg/g DW → 35 DAF: 116.89 ± 3.09 μg/g DW) and an 11.8% late-phase rise, accompanied by a 4.10-fold chlorophyll increase (15 DAF: 0.24 ± 0.01 mg/g DW → 35 DAF: 0.98 ± 0.02 mg/g DW) followed by rapid depletion (**Figure 2D**). Correlation analysis of vitamin E and chlorophyll components during seed development (**Supplementary Figure S3**) revealed a highly significant negative correlation across developmental stages (p< 0.001), with correlation intensity varying by genotype.

In summary, our findings delineate two distinct accumulation patterns of chlorophyll and tocopherol in YH and YL accessions: (1) the 45–55 DAF period emerged as a critical phase, during which high-VE genotypes sustained rapid tocopherol biosynthesis, whereas low-VE genotypes reached saturation with minimal subsequent accumulation (**Supplementary Figure S1**). (2) Using 35 DAF as a critical time point, chlorophyll exhibited rapid accumulation to peak levels alongside active tocopherol synthesis during the early phase (15–35 DAF), followed by chlorophyll degradation and sustained tocopherol accumulation to maximum levels in the late phase (35–50 DAF) (**Supplementary Figure S2**). These patterns suggest that chlorophyll- and tocopherol-synthetic genes are predominantly expressed during early seed development, while chlorophyll catabolic and tocopherol biosynthetic genes dominate metabolic processes in later stages. This temporal regulatory divergence provides crucial guidance for prioritizing candidate genes involved in metabolic flux redirection for seed vitamin E biosynthesis.

### 3.3 Transcriptome analysis of key genes involved in vitamin E accumulation during seeds development between YH and YL

#### 3.3.1 Transcriptome sequencing data collection

Based on the dynamic changes on seed tocopherol and chlorophyll contents during the whole seed developmental stages, we decided to conduct transcriptome analysis using YH and YL materials at five critical time points (15, 25, 35, 45 and 50 DAF). A total of 20 cDNA libraries were constructed with three biological replicates, yielding approximately 2,758,477,946 raw reads from a total of 60 samples. Quality assessment revealed an average Q30 score of 93.03%, indicating base call accuracy ≥99.9%. Between 91.93% and 94.75% of reads were uniquely mapped to the ZS11 reference genome (**Supplementary Table S3**).

Principal component analysis (PCA) demonstrated tight clustering between biological replicates for each time points, confirming good quality of sample collection (**Supplementary Figure S4**). Six differentially expressed genes (DEGs) were randomly selected for qRT-PCR to validate transcriptomic reliability, a strong concordance was acquired between RNA-seq and qRT-PCR, verifying the robustness of our transcriptomic dataset (**Supplementary Figure S5**).

#### 3.3.2 DEG gene analysis between YH and YL materials at different seed development stages

We identified 18,524; 15,355; 16,114; and 17,681 DEGs in YH01 during 15/25, 25/35, 35/45, and 45/50 DAF intervals, respectively. Corresponding DEG counts for YH07, YL02, and YL04 at these DAF intervals were 17,359; 14,557; 14,868; 19,303; 18,854; 13,548; 16,246; 12,807; 18,463; 16,687; 24,499; and 11,094, respectively (**Supplementary Table S4**).

Inter-accession comparisons between high- (YH) and low-VE (YL) materials revealed dynamic DEG profiles: 14,775; 16,515; 14,550; 16,568 DEGs were observed between YH and YL accessions at 15 DAF; 12,803; 14,628; 15,251; 17,243 at 25 DAF; 10,741; 11,043; 12,210; 12,136 at 35 DAF; 11,041; 18,576; 12,283; 18,931 at 45 DAF; and 10,989; 11,275; 15,178; 12,045 at 50 DAF (**Supplementary Table S4**). Significant temporal shifts in upregulated/downregulated gene ratios were observed across comparison groups.

**Figure 3:**
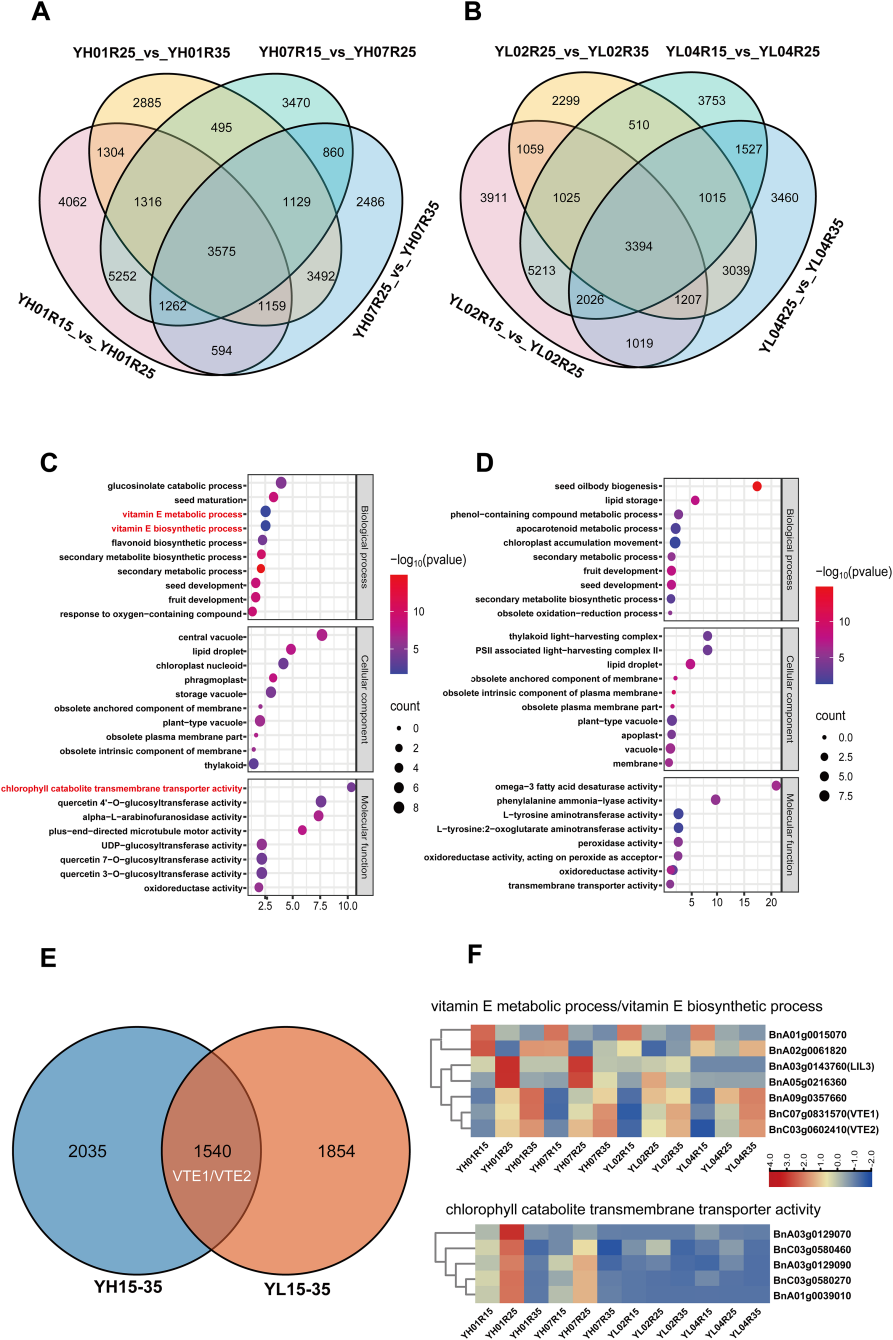
Analysis of differentially expressed genes (DEGs) between 15–35 DAF. **(A)** The Venn diagram of DEGs in YH01R15_VS_ YH01R25 ,YH01R25_VS_ YH01R35 and YH07R15_VS_ YH07R25 ,YH07R25_VS_ YH07R35. **(B)** The Venn diagram of DEGs in YL02R15_VS_ YL02R25 ,YL02R25_VS_ YL02R35 and YL04R15_VS_ YL04R25 ,YL04R25_VS_ YL04R35. **(C)** Gene Ontology (GO) analysis of 3575 DEGs at three developmental stages in YH. **(D)** Gene Ontology(GO)analysis of 3394 DEGs at three developmental stages in YL. **(E)** The Venn diagram of DEGs in YH15–35 and YL15-35. **(F)** Heat map of expression patterns of DEGs enriched into vitamin E metabolic pathway.

To identify conserved and divergent DEGs between high-/low-VE materials, we focused on two developmental windows (15–35 DAF and 35–50 DAF) based on chlorophyll-tocopherol correlations. During 15–35 DAF, 3,575 shared DEGs were identified in YH accessions (**Figure 3A**) versus 3,394 in YL (**Figure 3B**). Gene Ontology (GO) enrichment of these DEGs highlighted predominant pathways as following: transferase activity and peroxidase activity (molecular function); cytoplasm, plasma membrane, and vacuole (cellular component); secondary metabolic process and secondary metabolite biosynthesis (biological process) (**Figures 3C, D**). Notably, YH-specific enrichment during 15–35 DAF included three critical biological processes: vitamin E metabolic process, vitamin E biosynthetic process, and transmembrane transport of chlorophyll degradation products (**Figures 3C, D**), pathways unreported in YL. Literature-supported associations between these processes and tocopherol synthesis suggest their pivotal role in early-stage VE divergence. Specifically, 7 genes were enriched in vitamin E metabolism/biosynthesis pathways, and 5 genes in chlorophyll degradation product transport (**Table 1**).

**Table 1:** List of candidate genes related to vitamin E synthesis in early development of rapeseed (15–35 days).

Gene ID	Description
Vitamin E metabolic process/vitamin E biosynthetic process	
BnA01g0015070	Encodes cystine lyase which is expected to be involved in amino acid metabolism, providing the plant with cysteine and the generation of precursors of ethylene biosynthesis.
BnA02g0061820	Encodes a cytosolic tyrosine aminotransferase which is strongly induced upon aging and coronatine treatment.
BnA03g0143760(LIL3)	Encodes Lil3:1 (light-harvesting-like) protein. Belongs to the Lhc super-gene family encodes the light-harvesting chlorophyll a/b-binding (LHC) proteins that constitute the antenna system of the photosynthetic apparatus.
BnA05g0216360	Tyrosine transaminase family protein
BnA09g0357660	Encodes a C-S lyase involved in converting S-alkylthiohydroximate to thiohydroximate in glucosinolate biosynthesis.
BnC07g0831570(VTE1)	Tocopherol cyclase involved in tocopherol (vitamin E) synthesis. VTE1 over-expressing plants have increased tocopherol indicating VTE1 is a major limiting factor in tocopherol synthesis.
BnC03g0602410(VTE2)	Encodes homogentisate phytyltransferase involved in tocopherol biosynthesis.
Chlorophyll catabolite transmembrane transporter activity	
BnC03g0580270	encodes an ATP-dependent MRP-like ABC transporter able to transport glutathione-conjugates as well as chlorophyll catabolites.
BnA01g0039010	
BnC03g0580460	
BnA03g0129070	
BnA03g0129090	

Venn analysis identified 1,540 shared DEGs between high-/low-VE materials (**Figure 3E**). Among the 12 genes associated with the aforementioned GO terms, *BnC07g0831570* (*VTE1*) and *BnC03g0602410* (*VTE2*) were expressed in both groups, while 10 genes exhibited YH-specific expression (**Figure 3F**), potentially explaining enhanced VE accumulation in YH during early seed development.

**Figure 4:**
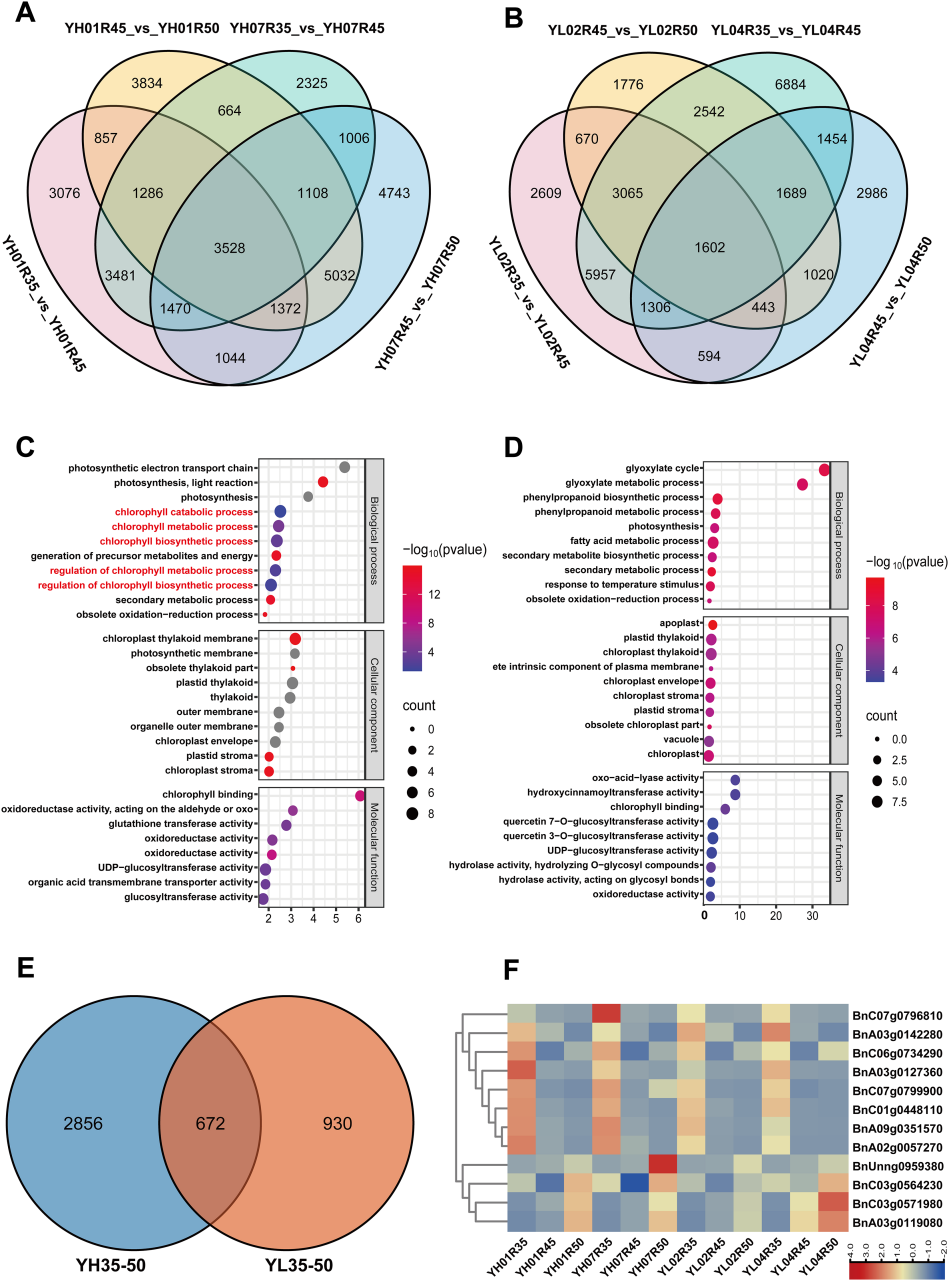
Analysis of differentially expressed genes (DEGs) between 35–50 DAF. **(A)** The Venn diagram of DEGs in YH01R35_VS_ YH01R45 ,YH01R45_VS_ YH01R50 and YH07R35_VS_ YH07R45 ,YH07R45_VS_ YH07R50. **(B)** The Venn diagram of DEGs in YL02R35_VS_ YL02R45 ,YL02R45_VS_ YL02R50 and YL04R35_VS_ YL04R45 ,YL04R45_VS_ YL04R50. **(C)** Gene Ontology (GO) analysis of 3528 DEGs at three developmental stages in YH. **(D)** Gene Ontology (GO) analysis of 1602 DEGs at three developmental stages in YL. **(E)** The Venn diagram of DEGs in YH35–50 and YL35-50. **(F)** Heat map of expression patterns of DEGs enriched into chlorophyll synthesis and metabolic pathways.

During 35–50 DAF, 3,528 shared DEGs were detected in YH (**Figure 4A**) versus 1,602 in YL (**Figure 4B**), indicating intensified transcriptional regulation in high-VE materials. YH-specific GO enrichment featured five chlorophyll-related processes: chlorophyll metabolic process, chlorophyll biosynthetic process, chlorophyll catabolic process, regulation of chlorophyll biosynthesis, and regulation of chlorophyll metabolism (**Figure 4C**; **Supplementary Table S5**). These pathways were absent in YL (**Figure 4D**).

Functional annotation of pathway-associated genes identified 24 candidates involved in these biological processes. Venn diagram analysis (**Figure 4E**) comparing their expression profiles between high- and low-VE materials revealed 19 genes exclusively upregulated in YH-derived DEGs, while 5 genes were specific to YL accessions. This indicates heightened engagement of vitamin E-related pathways in YH genotypes. Among these, 12 chlorophyll metabolism-associated genes exhibited coordinated expression patterns, with significantly elevated transcript levels in YH compared to YL (**Figure 4F**). Functional annotations of these 12 genes were shown in **Table 2**. We hypothesize that these genes, particularly those governing chlorophyll-to-tocopherol metabolic flux redirection, constitute the primary molecular basis for divergent tocopherol accumulation between high- and low-VE materials during late seed development.

**Table 2:** List of candidate genes related to chlorophyll metabolism in the late stage of rapeseed development (35–50 days).

Gene ID	Description
BnC03g0571980(SGR2/NYE2)	Acts antagonistically with SGR1 to balance chlorophyll catabolism in chloroplasts with the dismantling and remobilizing of other cellular components in senescing leaf cells.
BnA03g0119080(SGR2/NYE2)	Acts antagonistically with SGR1 to balance chlorophyll catabolism in chloroplasts with the dismantling and remobilizing of other cellular components in senescing leaf cells.
BnA09g0351570(HEMA1)	Encodes a protein with glutamyl-tRNA reductase (GluTR) activity, catalyzing the NADPH-dependent reduction of Glu-tRNA(Glu) to glutamate 1-semialdehyde (GSA) with the release of free tRNA(Glu). It is involved in the early steps of chlorophyll biosynthesis.
BnC07g0796810(GLK2)	Encodes GLK2, Golden2-like 2, one of a pair of partially redundant nuclear transcription factors that regulate chloroplast development in a cell-autonomous manner. GLK1, Golden2-like 1, is encoded by At2g20570. GLK1 and GLK2 regulate the expression of the photosynthetic apparatus.
BnA02g0057270(GLK2)	Encodes GLK2, Golden2-like 2, one of a pair of partially redundant nuclear transcription factors that regulate chloroplast development in a cell-autonomous manner. GLK1, Golden2-like 1, is encoded by At2g20570. GLK1 and GLK3 regulate the expression of the photosynthetic apparatus.
BnA03g0142280(DXS)	Encodes a protein with 1-deoxyxylulose 5-phosphate synthase activity involved in the MEP pathway. It is essential for chloroplast development in Arabidopsis
BnC01g0448110(DXS)	Encodes a protein with 1-deoxyxylulose 5-phosphate synthase activity involved in the MEP pathway. It is essential for chloroplast development in Arabidopsis
BnC03g0564230(NTRC)	Encodes a NADPH thioredoxin reductase involved in chloroplast protection against oxidative damage.
BnC07g0799900(LIL3)	Encodes a light-harvesting-like protein that is involved in chlorophyll and tocopherol biosynthesis anchoring geranylgeranyl reductase in the thylakoid membrane.
BnA03g0127360(GUN4)	GUN, genomes uncoupled, is necessary for coupling the expression of some nuclear genes to the functional state of the chloroplast. Binds to the magnesium chelatase complex and promotes formation of the substrate,a tetrapyrrole signaling molecule. Porphyrin-binding protein that enhances the activity of Mg-chelatase. Although required for chlorophyll accumulation under normal growth conditions, GUN4 is not essential for chlorophyll synthesis.
BnC06g0734290(CAO)	Encodes chlorophyllide a oxygenase which converts chlorophyllide a to chlorophyllide b by catalyzing two successive hydroxylations at the 7-methyl group of chlorophyllide a.
BnUnng0959380(GATA22)	Encodes a member of the GATA factor family of zinc finger transcription factors. Modulate chlorophyll biosynthesis and glutamate synthase (GLU1/Fd-GOGAT) expression.

### 3.4 Weighted gene co-expression network analysis and visualization

**Figure 5:**
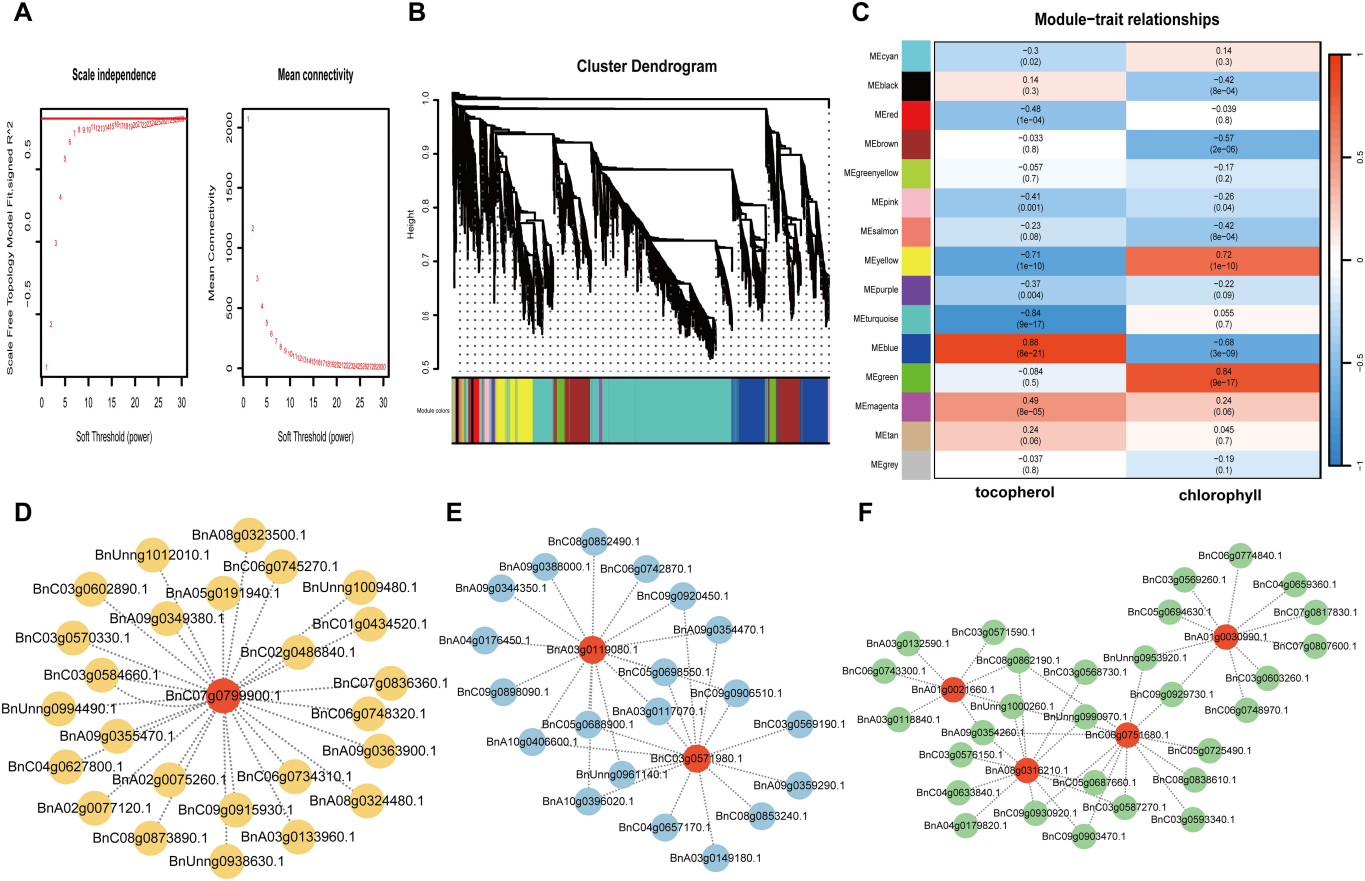
Weighted gene co-expression network analysis (WGCNA) of DEGs identified during the development process of rapeseed seeds with high and low levels of tocopherols. **(A)** Soft threshold selection diagram (red horizontal line indicates R2 = 0.8). **(B)** Module hierarchical clustering tree showing 15 modules of co-expressed genes. **(C)** Correlation between module and tocopherol content and chlorophyll content and its corresponding P value (in parentheses). **(D)** Co-expression network of key genes from “yellow” module. **(E)** Co-expression network of key genes from “blue” module. **(F)** Co-expression network of key genes from “Turquoise” module.

To gain further insight into the regulation of Chlorophyll metabolism and tocopherol biosynthesis, we carried out WGCNA analysis to investigate the co-expression gene modules and the critical hub genes. Genes with correlation coefficients >0.8 that conformed to the scale-free network distribution were clustered into distinct modules using a soft thresholding power of 12 (**Figure 5A**). A total of fifteen co-expression modules were identified (**Figure 5B**). Subsequent module-trait association analysis revealed four critical modules (designated as MEyellow, MEblue, MEturquoise, and MEgreen) exhibiting statistically significant correlations with chlorophyll and tocopherol content (correlation coefficient >0.5, P<0.05) (**Figure 5C**). The MEyellow module exhibited a strong negative correlation with tocopherol content (r = −0.71) and a positive correlation with chlorophyll content (r = 0.72), encompassing 444 genes. Conversely, the MEblue module showed a highly positive correlation with tocopherol (r = 0.88) but a negative correlation with chlorophyll (r = −0.68), containing 828 genes. The MEturquoise module was positively associated with chlorophyll content (r = 0.84) including 2,305 genes, while the MEgreen module displayed a strong positive correlation with tocopherol (r = 0.84; 205 genes) (**Supplementary Table S6**). Further analysis demonstrated significant overlap in DEGs enrichment among MEyellow, MEblue, and MEturquoise modules, suggesting their synergistic roles in chlorophyll metabolism. Within MEyellow, the hub gene LIL3 (*BnC07g0799900*) exhibited the highest network connectivity (**Figure 5D**). This gene encodes a light-harvesting-like complex protein that anchors geranylgeranyl reductase (GGDR) to thylakoid membranes, critically coordinating chlorophyll and tocopherol biosynthesis (Peltier et al., 2004). Its centrality in the network implies a regulatory mechanism balancing photosynthetic pigment metabolism and vitamin E precursor synthesis. In MEblue, two paralogs of SGR2/NYE2 (*BnA03g0119080*, *BnC03g0571980*) were identified as key nodes (**Figure 5E**). These genes form an antagonistic function with SGR1, balancing chlorophyll degradation and cellular component recycling during senescence (Ono et al., 2019; Yamatani et al., 2022). The MEturquoise module harbored four functionally central genes (**Figure 5F**), PPD (*BnA01g0021660*), a multifunctional esterase modulating phyto hormone precursors (e.g., methyl IAA and methyl jasmonate) (Yang et al., 2008); ELIP1 (*BnA01g0030990*), an early light-induced protein involved in photo-protection and thylakoid complex assembly (Beck et al., 2017); and CRD1 (*BnA08g0316210/BnC06g0751680*), a ZIP-family metal transporter essential for chlorophyll degradation regulation (Hauenstein et al., 2022). Collectively, these hub genes constitute a coordinated network that synchronizes chlorophyll turnover and substrate flux equilibrium for vitamin E biosynthesis.

### 3.5 Candidate gene mapping for tocopherol biosynthesis regulation using BSA-seq

#### 3.5.1 Analysis of seed tocopherol contents in DH population

**Figure 6:**
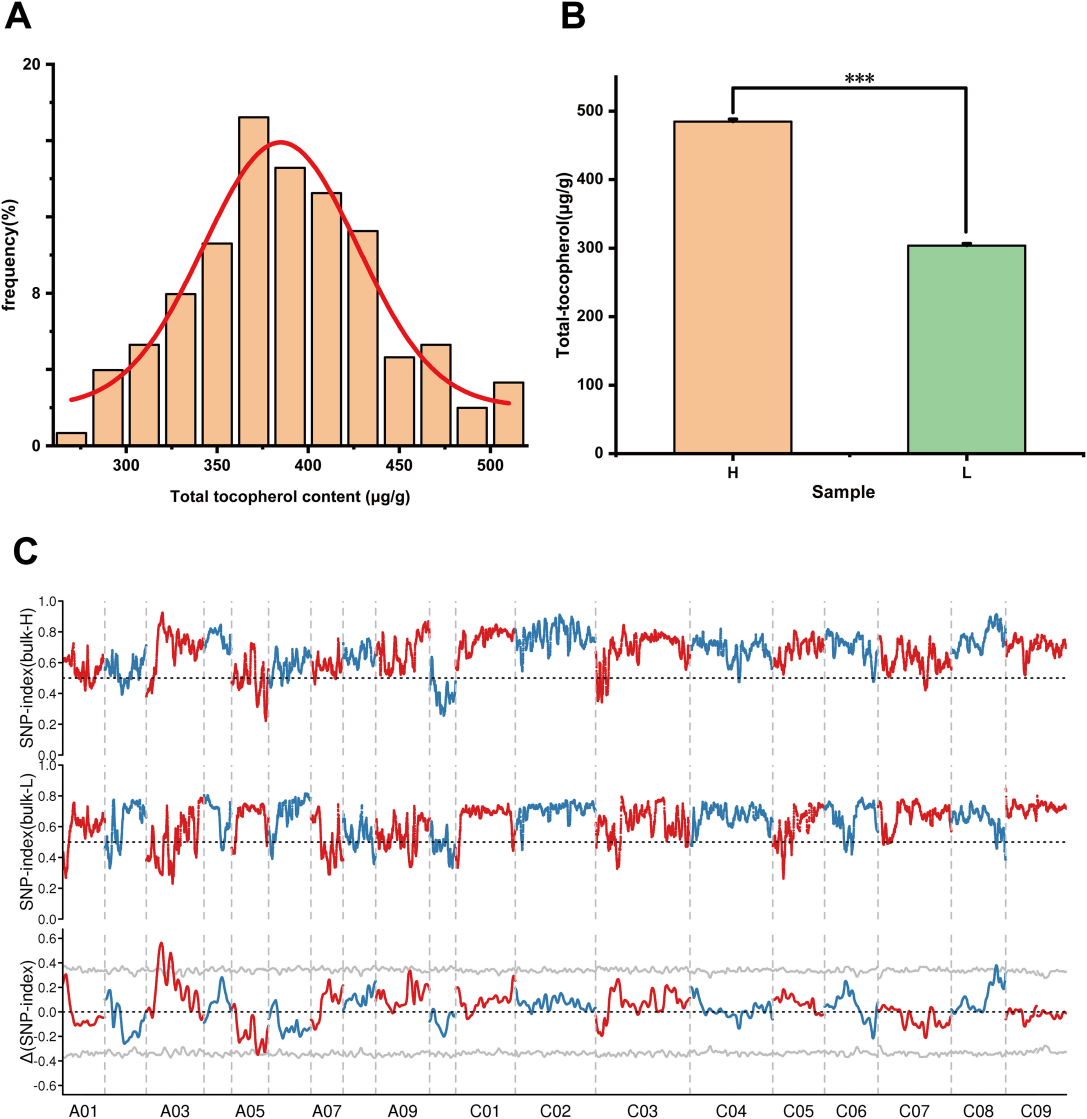
BSA-seq analysis. **(A)** Tocopherol content frequency distribution map of F_2_ population in the field. **(B)** The Performance of seed tocopherol content in two extreme mixed pools. Asterisks on the lines indicate significant differences between the mean samples by Student’s t-test (***P<0.001). **(C)** Distribution of SNP index association values on chromosomes, where the grey line represents the threshold (p = 0.001). Bars indicate means ± SE (n = 20).

Using YH07 and YL02 as parental lines, we constructed a mapping population consisting of 200 doubled haploid (DH) lines. The tocopherol content in mature seeds was quantitatively measured, and the histogram revealed a normal distribution pattern (**Figure 6A**). This indicates that seed tocopherol content in rapeseed represents a quantitative trait susceptible to environmental influences, while being potentially governed by major-effect genetic determinants. Subsequently, we ranked the DH lines based on tocopherol levels and established two extreme pools by seed vitamin E contents, selecting the top 20 and bottom 20 lines for high- and low-tocopherol bulk pools, respectively. The high- bulk pool (H) exhibited a 1.6-fold greater mean tocopherol content compared to the low- bulk pool (L) (**Figure 6B**). These pools were used for genetic mapping of key candidate genes regulating tocopherol biosynthesis in *Brassica napus*.

#### 3.5.2 BSA-Seq based mapping genes involved in tocopherol biosynthesis

Three DNA samples, including the parental line YH07 and the extreme H and L bulk populations, were subjected to sequencing. After quality filtering, 125 Gb of high-quality clean data were obtained. Sequencing data quality was rigorously evaluated. Quality metrics revealed GC content ranging from 37.93% to 38.55%, along with high sequencing accuracy (**Supplementary Table S7**). Reads were aligned to the *Brassica napus* cv. ZS11 reference genome (available at https://yanglab.hzau.edu.cn/BnIR/germplasm_info?id=ZS11.v2), achieving an average mapping rate of 93.48%, mean sequencing depth of 27.02×, and genome coverage of 64.75%. We identified 1,187,485 high-confidence variants, comprising 992,356 SNPs and 195,129 InDels in between H- and L- bulk pools (**Supplementary Table S8**). Variant detection was performed using GATK (version 3.7), and genomic distribution was analyzed with 100-kb sliding windows. Three candidate genomic regions were identified: two intervals spanning 11.54–14.66 Mb and 19.06–23.06 Mb on chromosome A03, and one interval at 37.19–39.13 Mb on chromosome C08 (**Figure 6C**).

### 3.6 Prediction of candidate genes involved in tocopherol biosynthesis using RNA-seq, BSA-seq and WGCNA

**Figure 7:**
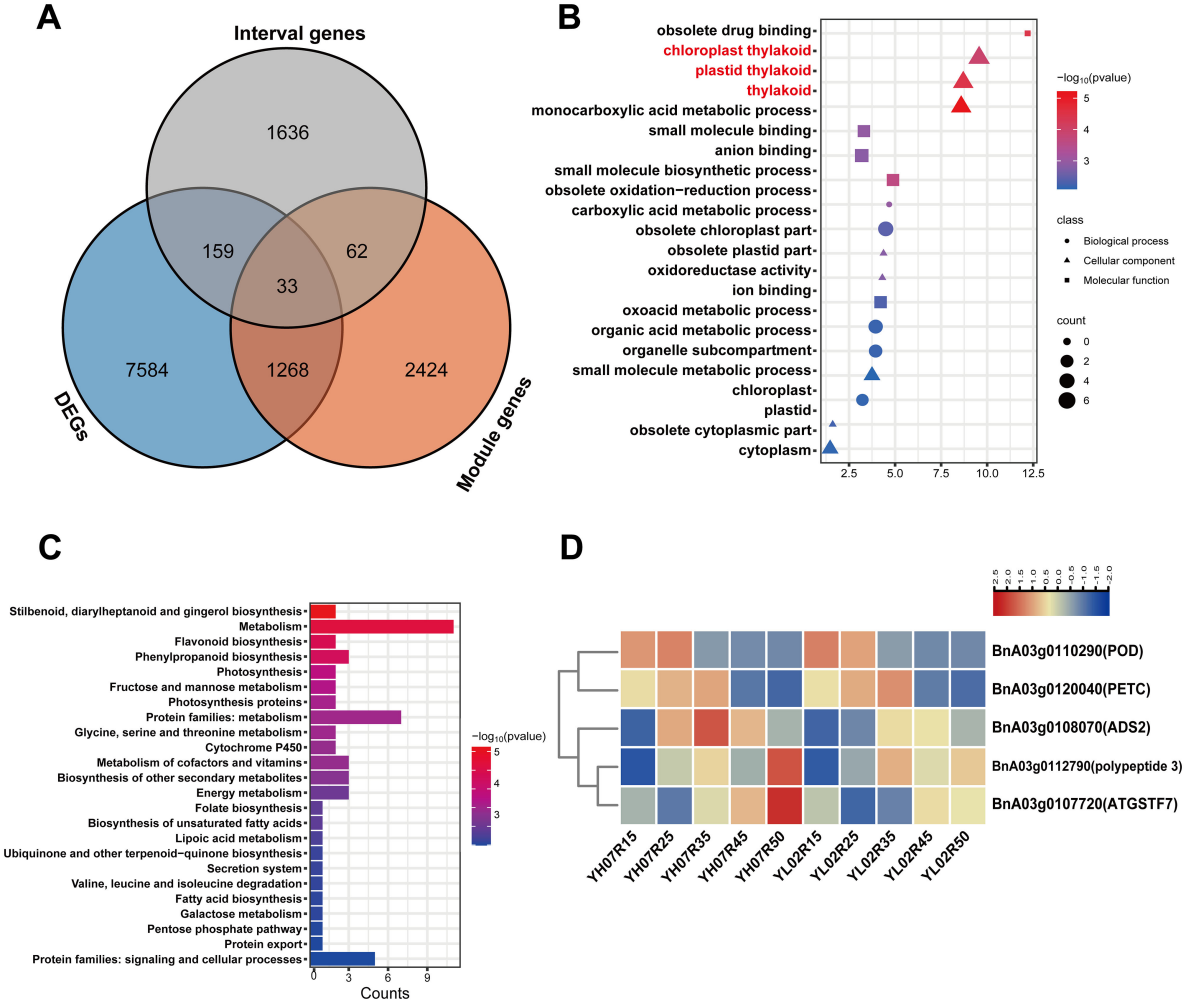
Combined Analysis of BSA-Seq and RNA-Seq. **(A)** The Venn diagram of DEGs in RNA-Seq,Module gene (WGCNA) and Interval gene (BSA-Seq). **(B)** GO analysis of common DEGs, the ordinate is the pathway and the abscissa is the gene ratio (the redder the color, the smaller the p value)). **(C)** KEGG analysis of common DEGs, the ordinate is the pathway and the abscissa is the gene ratio (the redder the color, the smaller the pvalue) **(D)** Heat map of expression patterns of DEGs enriched in thylakoid. The relative expression levels of DEGs were calculated using the Log2.

We integrated three datasets: 1,890 interval genes derived from BSA-Seq, 9,044 differentially expressed genes (DEGs) identified via RNA-Seq, and 3,782 module-associated genes obtained from WGCNA, a total of 33 co-overlapping genes were identified across these datasets (**Figure 7A**). Gene Ontology (GO) enrichment analysis of these 33 DEGs demonstrated significant enrichment in chloroplast thylakoid, plastid thylakoid, and thylakoid membrane-related terms (**Figure 7B**). KEGG pathway analysis of the DEGs revealed significant enrichment in biological pathways including “dibenzofuran, diarylheptanoid, and gingerol biosynthesis,” “metabolic pathways,” “flavonoid biosynthesis,” and “phenylpropanoid biosynthesis” (**Figure 7C**). These pathways are associated with antioxidative, anti-inflammatory, and antimicrobial activities, sharing precursor metabolites with tocopherol biosynthesis. By cross-referencing genes within these enriched pathways with candidate intervals identified through BSA-Seq, five overlapping genes were prioritized (**Figure 7D**): *BnA03g0107720* encodes a glutathione S-transferase, potentially modulating tocopherol biosynthesis via redox homeostasis and detoxification processes. *BnA03g0120040* encodes the Rieske FeS center of the cytochrome b6f complex, regulating photosynthetic electron transport, which may indirectly influence tocopherol synthesis. *BnA03g0110290* belongs to the peroxidase superfamily, likely maintaining reactive oxygen species (ROS) levels critical for sustaining normal tocopherol biosynthesis. *BnA03g0108070* is implicated in seed lipid biosynthesis, with evidence suggesting shared regulatory mechanisms between tocopherol and fatty acid biosynthesis in soybean (Chu et al., 2023). *BnA03g0112790* encodes coumarate 3-hydroxylase (C3H), involved in lignin and flavonoid biosynthesis, pathways that share precursors with tocopherol metabolism. Heatmap of these five genes demonstrated differential expression in both high- and low-VE materials (**Figure 7D**), highlighting their function at different stage of seed development for tocopherol biosynthesis.

## 4 Discussion

This study was designed to elucidate the synergistic regulatory network between chlorophyll degradation and vitamin E (tocopherol) biosynthesis during seed development in Brassica napus L. by integrating bulk segregant analysis sequencing (BSA-Seq) and RNA-sequencing (RNA-Seq) technologies. The objectives focused on clarifying the spatiotemporal coupling of chlorophyll synthesis/degradation and tocopherol biosynthetic pathways during seed maturation, identifying core candidate genes and regulatory modules governing these metabolic processes, and establishing a theoretical foundation with molecular targets for synchronously improving rapeseed oil quality (via chlorophyll residue reduction) and nutritional value (through tocopherol content enhancement). While the multi-omics integration revealed a coordinated regulatory framework linking chlorophyll and tocopherol metabolism, further experimental validation is required to decipher mechanistic causality, and expanded investigations into genotype-environment interactions to translate these findings into practical breeding applications.

### 4.1 Tocopherol variation in *Brassica napus* seeds

**Figure 8:**
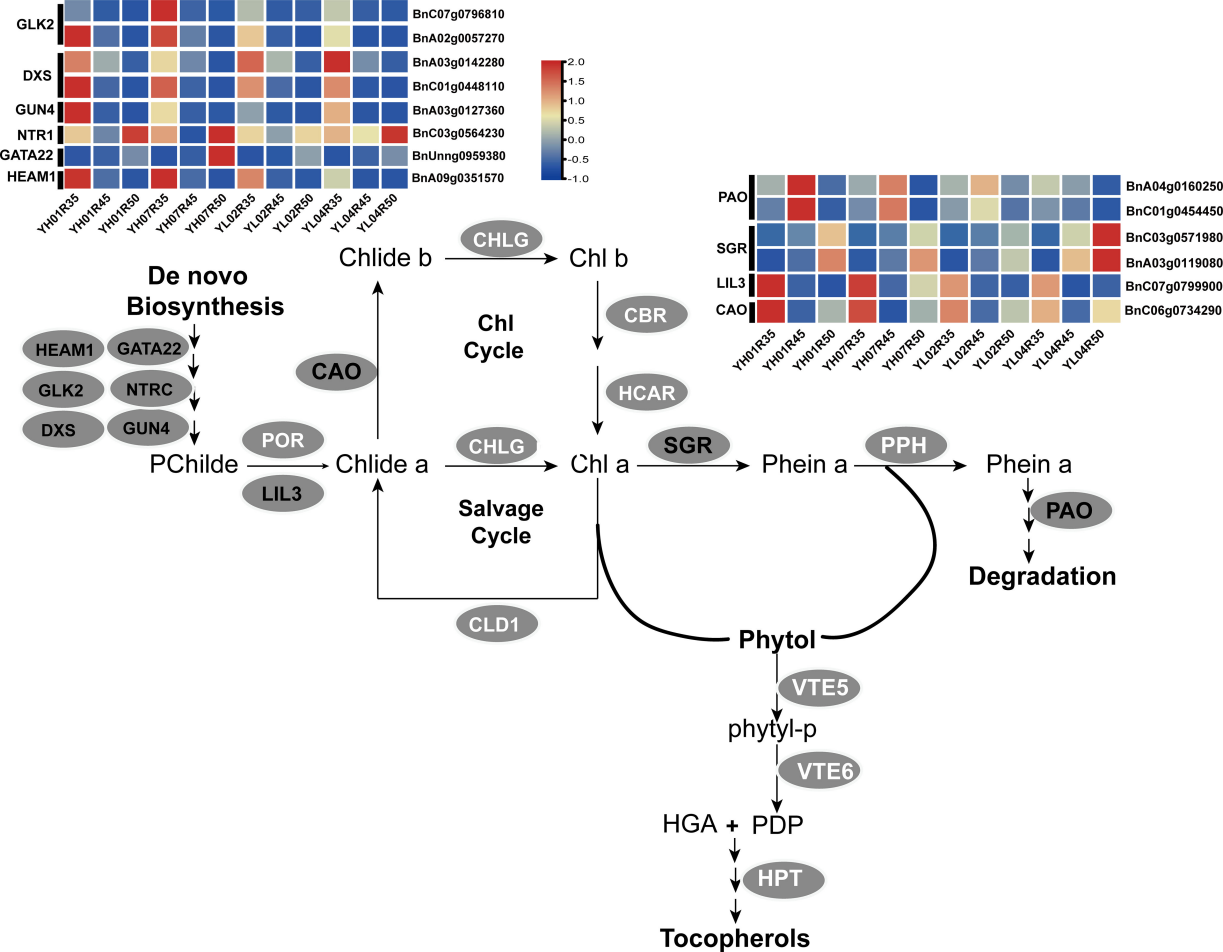
Analysis of the expression levels of candidate genes in the chlorophyll metabolic pathway. The candidate genes selected in this study are highlighted in red text, while other key pathway genes are highlighted in white text. GLK2, golden2-like2; DXS,1-deoxy-D-xylose-5-phosphate synthase; GUN4, genomes uncoupled 4; NTRC, NADPH-dependent thioredoxin reductase; HEMA1, glutamyl-tRNA reductase1; PAO, pheophorbide a oxygenase; SGR, stay-green; LIL3, light-harvesting-like3; CAO, chlorophyllide an oxygenase; PPH, pheophytynase; POR, protochlorophyllide an oxidoreductase; CHLG, chlorophyll synthase; CLD1, chlorophyll dephytylase1; CBR, Chlorophyll b reductase; HCAR: 7-hydroxymethyl chlorophyll a reductase.

As a vital oilseed crop of significant economic importance in vegetable oil production, *Brassica napus* seeds are also rich in vitamin E (tocopherols), which offers considerable nutritional value for human health. Through comparative analysis of high- and low-vitamin E genetic materials, we revealed stage-specific regulatory mechanisms governing tocopherol biosynthesis. The differential expression of core VE biosynthetic genes (*LIL3*, *VTE1*, *and VTE2*) primarily explained the initial divergence in tocopherol content during early seed development; while in later maturation stages (35–50 DAF), the accelerated VE accumulation in high-VE materials correlated with enhanced chlorophyll degradation marked by upregulation of chlorophyll catabolism genes (*NYC1*, *SGR*, *PPH*) (**Figure 8**). This metabolic shift likely facilitates phytol precursor mobilization for tocopherol biosynthesis, establishing a novel linkage between chloroplast senescence and vitamin E accumulation in oilseeds. Notably, our dynamic analysis across seven developmental stages uncovered previously unrecognized metabolic coordination between chlorophyll degradation and tocopherol biosynthesis.

### 4.2 Potential interrelationship between vitamin E and chlorophyll during seed development

Vitamin E, as a crucial lipid-soluble antioxidant, exhibits tissue-specific accumulation patterns that are significantly regulated by developmental stages and genetic backgrounds (Sattler et al., 2004). To elucidate the molecular mechanisms underlying vitamin E biosynthesis and accumulation in *Brassica napus* L. seeds, this study selected two groups of *B. napus* germplasms (YH01/YH07 and YL02/YL04) with contrasting vitamin E contents for temporal analysis during seed development. Results demonstrated a continuous accumulation of total vitamin E content throughout seed development, stabilizing at physiological maturity (**Supplementary Figure S1**). Compositional profiling revealed γ-tocopherol as the predominant isoform, followed by α-tocopherol, with δ-tocopherol exhibiting the lowest proportion, consistent with the majority of angiosperm seeds (Mène-Saffrané and Pellaud, 2017). Notably, significant divergence in accumulation kinetics between high- and low-vitamin E materials ware observed at critical developmental phases (particularly 35–50 days after flowering, DAF), providing pivotal temporal windows for subsequent identification of key regulatory genes (**Figure 2**). The spatiotemporal coupling between chlorophyll metabolism and vitamin E biosynthesis during seed maturation warrants attention. This study identified a biphasic dynamic pattern in chlorophyll a/b content characterized by initial accumulation followed by degradation, while vitamin E biosynthesis intensified markedly during late seed development (35–50 DAF), suggesting potential metabolic interactions (**Figure 2**). Early-stage chlorophyll biosynthesis likely supplies phytol precursors for tocopherol synthesis, whereas chloroplast-to-storage organelle transformation during maturation facilitates phytol release via chlorophyll catabolism, thereby promoting tocopherol biosynthesis. Intriguingly, a contrasting correlation pattern emerged compared to citrus fruit development studies (Zhao et al., 2021) reported significant positive correlations between α-tocopherol and chlorophyll in young citrus fruits, whereas our data revealed negative trends during seed maturation(**Supplementary Figure S3**). This discrepancy may originate from organ-specific metabolic regulation, where early fruit development prioritizes photosynthetic organelle maintenance, whereas seed maturation necessitates programmed chloroplast dismantling to optimize storage compound accumulation.

### 4.3 Impact of Vitamin E biosynthesis-related genes on vitamin E accumulation during early seed development in *Brassica napus*

By synergizing transcriptomic and genetic mapping data, we successfully identified key genes associated with tocopherol accumulation, providing a strategic foundation for subsequent functional genomic investigations. The expression of vitamin E biosynthesis genes effectively regulates vitamin E accumulation during seed development. This investigation identified that early seed development (15–35 DAF) involves polygenic coordination in vitamin E accumulation. Through systematic analysis, 12 core pathway genes were screened (**Table 1**), whose expression profiles exhibited significant correlations with vitamin E accumulation dynamics. Notably, this study pioneers the characterization of dynamic expression patterns of *VTE1* and *VTE2* genes during early seed development in *Brassica napus* L. (**Figure 3F**). The core biosynthetic genes *BnC07g0831570* (*VTE1*) and *BnC03g0602410* (*VTE2*), encoding tocopherol cyclase and homogentisate phytyltransferase respectively, have been previously validated as rate-limiting enzymes in the vitamin E pathway in Arabidopsis (Porfirova et al., 2002; Collakova and DellaPenna, 2003a, b). These findings suggest their central regulatory roles in vitamin E biosynthesis in rapeseed. Additionally, BnA03g0143760 was annotated as a LIL3.1 protein, belonging to the light-harvesting chlorophyll a/b-binding (Lhc) protein superfamily. Previous studies indicate that LIL3 functionally participates in both chlorophyll and tocopherol biosynthesis by stabilizing geranylgeranyl reductase (Tanaka et al., 2010; Mork-Jansson and Eichacker, 2018). This study first reports the seed-specific upregulation of *LIL3.1* expression during early seed development in *B. napus*. We hypothesize that this gene may indirectly modulate vitamin E biosynthesis via chlorophyll precursor homeostasis. In chlorophyll catabolite transmembrane transport pathways, five AB3C transporter family genes were identified (**Figure 3**). This family has been implicated in glutathione conjugate and chlorophyll breakdown product translocation (Verrier et al., 2008). Strikingly, these transporter genes exhibited significantly higher expression levels in high-vitamin E (YH) accessions compared to low-vitamin E (YL) counterparts, with pronounced upregulation during the rapid vitamin E accumulation phase (15–35 DAF) (**Figure 3**). This suggests that metabolites from chlorophyll degradation may be recycled as vitamin E precursors via specialized transport systems, and inter-accession variation in transport efficiency could critically influence vitamin E accumulation. These observations align with the recently proposed “chlorophyll-tocopherol metabolic coupling” phenomenon in oil crops, where phytol recycling for tocopherol biosynthesis and homeostasis involves coordinated spatiotemporal expression of multiple ABH family members (Bao et al., 2025). However, precise mechanistic insights require further validation.

### 4.4 Influence of chlorophyll metabolism-related genes on vitamin E accumulation during late seed development in *Brassica napus*

Recent studies have elucidated that phytol diphosphate (PDP), the precursor for tocopherol biosynthesis in seeds, predominantly originates from chlorophyll degradation pathways. Specifically, free phytol released during chlorophyll catabolism undergoes two-step phosphorylation reactions mediated by *VTE5* and *VTE6*, ultimately entering the tocopherol biosynthesis pathway (Vom Dorp et al., 2015; Gutbrod et al., 2019). Previous work from our laboratory demonstrated that the expression level of the chlorophyll synthase gene *CHLG* in Arabidopsis exhibits a significant positive correlation with tocopherol synthesis efficiency (Zhang et al., 2015). Furthermore, through *CHLSYN* suppression and HGA (homogentisate) biosynthesis enhancement, we successfully elevated total tocopherol content in green oilseed crops (Qin et al., 2024). Notably, although the functional roles of chlorophyll degradation enzymes (e.g., CLH1, CLH2, PPH, NYE1, and NYE2) have been systematically characterized in Arabidopsis, the phytol hydrolase directly linking chlorophyll degradation to tocopherol biosynthesis in seeds remains unidentified (Zhang et al., 2014).

This study revealed through integrated multi-omics analysis that high-vitamin E accessions (YH) markedly activated chlorophyll metabolism and translocation-related pathways during mid-late seed developmental stages. A total of 24 key regulatory genes were identified, including 12 core genes directly associated with chlorophyll metabolism (**Table 2**). Transcriptomic profiling (**Figure 8**) demonstrated significant upregulation of *BnA04g0160250* and *BnC01g0454450*, encoding pheophorbide an oxygenase (PAO), in YH accessions. As a pivotal enzyme in the canonical chlorophyll catabolic pathway, PAO upregulation likely accelerates chlorophyll degradation to promote free phytol release, thereby supplying precursors for tocopherol biosynthesis (Ghandchi et al., 2016). Notably, *SGR2*/*NYE2* homologs (*BnC03g0571980* and *BnA03g0119080*) exhibited pronounced overexpression in low-tocopherol accessions (YL04) (**Figure 8**), suggesting divergent regulatory roles of this gene family across genetic backgrounds.

Further analysis revealed that high expression of *LIL3* (*BnC07g0799900*) in YH accessions may synergistically enhance chlorophyll biosynthesis and tocopherol production by anchoring geranylgeranyl reductase (GGR) to the thylakoid membrane (Tanaka et al., 2010). Concurrently, upregulation of chlorophyll a oxygenase (CAO, BnC06g0734290) likely modulates chlorophyll b synthesis to dynamically balance chlorophyll cycling and tocopherol precursor supply (Dey et al., 2023). Furthermore, coordinated overexpression of chloroplast development regulators including HEMA1 (*BnA09g0351570*), GATA22 (*BnUnng0959380*), GLK2(*BnC07g0796810*/*BnA02g0057270*), NTRC (*BnC03g0564230*), and GUN4 (*BnA03g0127360*) corroborates enhanced chloroplast metabolic network activity in YH materials (Bastakis et al., 2018; Zhi et al., 2019; Wittmann et al., 2023).

Additionally, elevated expression of DXS (*BnC01g0448110*/*BnA03g0142280*), a key methylerythritol phosphate (*MEP*) pathway gene, may synergistically boost chloroplast development and tocopherol biosynthesis through augmented isoprenoid precursor provision (de Luna-Valdez et al., 2021).

In summary, our study systematically elucidates a trilateral synergistic mechanism encompassing “chlorophyll degradation–precursor provisioning–metabolic regulation” in high-vitamin E *Brassica napus* accessions. This integrated framework operates by accelerating precursor mobilization through upregulated catabolic genes such as *PAO* to enhance chlorophyll-derived phytol supply, maintaining metabolic homeostasis mediated by *LIL3* and *CAO* to stabilize chloroplast functionality, and orchestrating high-efficiency metabolic networking via regulatory genes like *GLK2* and *GUN4* to optimize biosynthesis flux. Notably, *LIL3* demonstrates dual-phase regulatory roles, participating in early-stage vitamin E biosynthesis and coordinating programmed chloroplast dismantling during maturation, which suggests its pivotal function in spatiotemporal coordination of these processes. This multi-tiered regulatory framework provides novel theoretical foundations and genetic resources for vitamin E biofortification in oilseed crops.

## 5 Conclusions

This study elucidated the molecular mechanisms underlying dynamic vitamin E accumulation during seed development in *Brassica napus* through the transcriptomic analysis, establishing for the first time the temporal regulatory characteristics of vitamin E biosynthesis and chlorophyll metabolism pathways across distinct developmental phases. Comparative analysis of high- and low-vitamin E accessions revealed a significant negative correlation between vitamin E accumulation and chlorophyll degradation, while proposing a stage-specific regulatory model governed by transcriptional networks: During early development (15–35 DAF), key biosynthetic genes (*VTE1*, *VTE2*) were markedly upregulated, accompanied by potential precursor transport facilitation through ABCC transporter family members to promote tocopherol synthesis. In mid-late stages (35–50 DAF), coordinated activation of chlorophyll catabolism genes (*SGR*, *PAO*) with light-signaling regulators (LIL3, GLK2) established an efficient chlorophyll metabolic network that ultimately enhanced tocopherol production. BSA-Seq analysis further identified five core regulatory genes (*BnA03g010772*, *BnA03g0120040*, etc.) within the candidate region on chromosome A03, which exert direct or indirect regulatory effects on tocopherol biosynthesis. These findings provide critical insights into the temporal coordination of vitamin E metabolism and chloroplast remodeling during seed maturation, offering valuable molecular targets for genetic enhancement of vitamin E content in oilseed crops.

## Data Availability

The RNAseq data analyzed for this study can be found here: National Center for Biotechnology Information (NCBl) BioProject database under accession number PRJNA1252958.
