# Supplementary material for: Dynamic identification of candidate genes associated with higher tocopherol biosynthesis in Brassica napus seeds
Source: Front Plant Sci. 2025 Jun 5;16:1613360. doi: 10.3389/fpls.2025.1613360 (PMC12176815; doi:10.3389/fpls.2025.1613360)
Supplement: Supplementary file 1 [file DataSheet1.docx]

Supplementary Material

# Supplementary Data

# Supplementary Figures and Tables

## Supplementary Figures


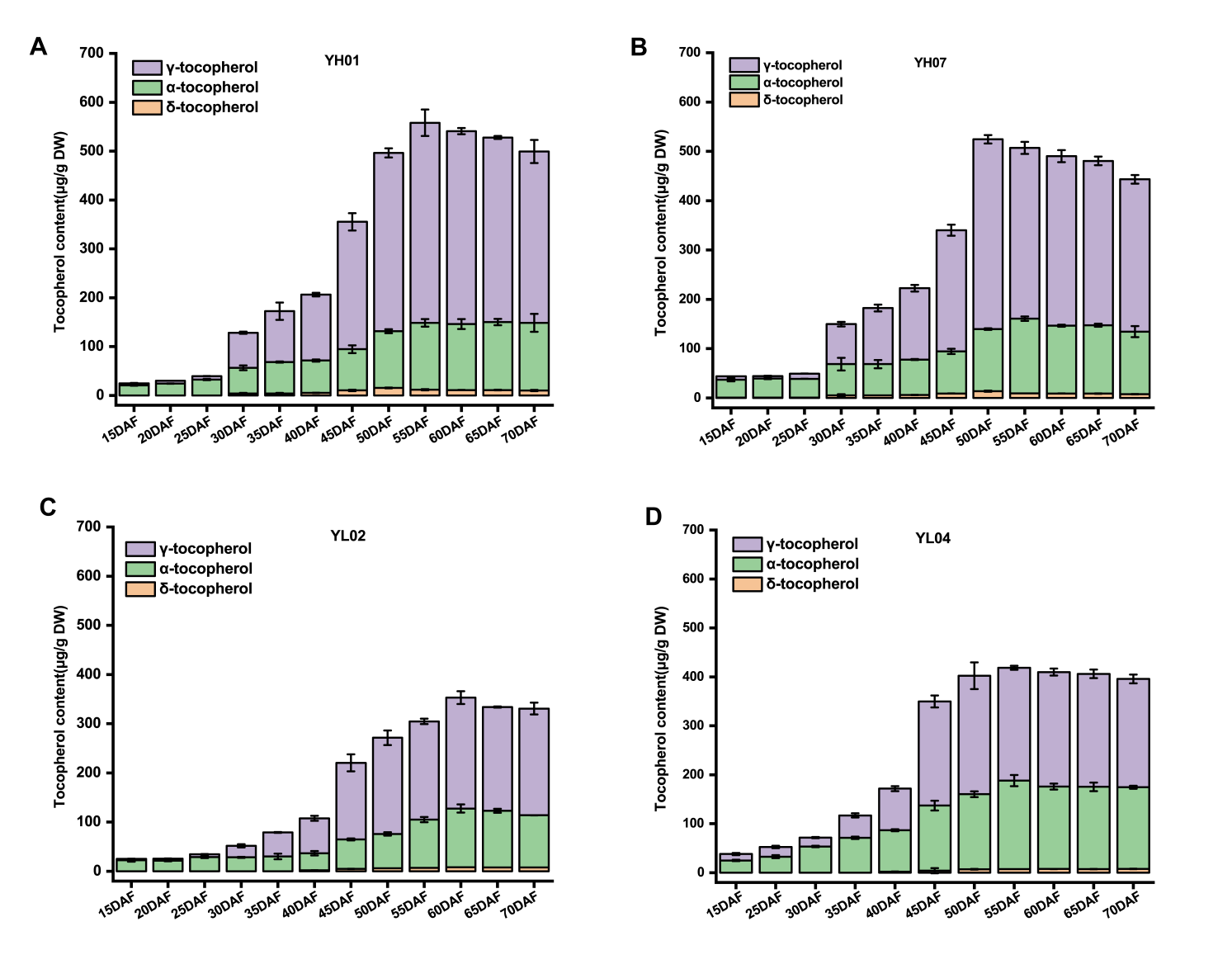


**Supplementary Figure S1.** Dynamic changes of tocopherol content in rapeseed every 5 days starting from 15 days after flowering.

(A) Dynamic changes of tocopherol content in YH01 rapeseed every 5 days starting from 15 days after flowering. (B) Dynamic changes of tocopherol content in YH07 rapeseed every 5 days starting from 15 days after flowering. (C) Dynamic changes of tocopherol content in YL02 rapeseed every 5 days starting from 15 days after flowering. (D) Dynamic changes of tocopherol content in YL04 rapeseed every 5 days starting from 15 days after flowering. The results of each material are based on three biological and three technical replicates. The error bars indicate standard errors.


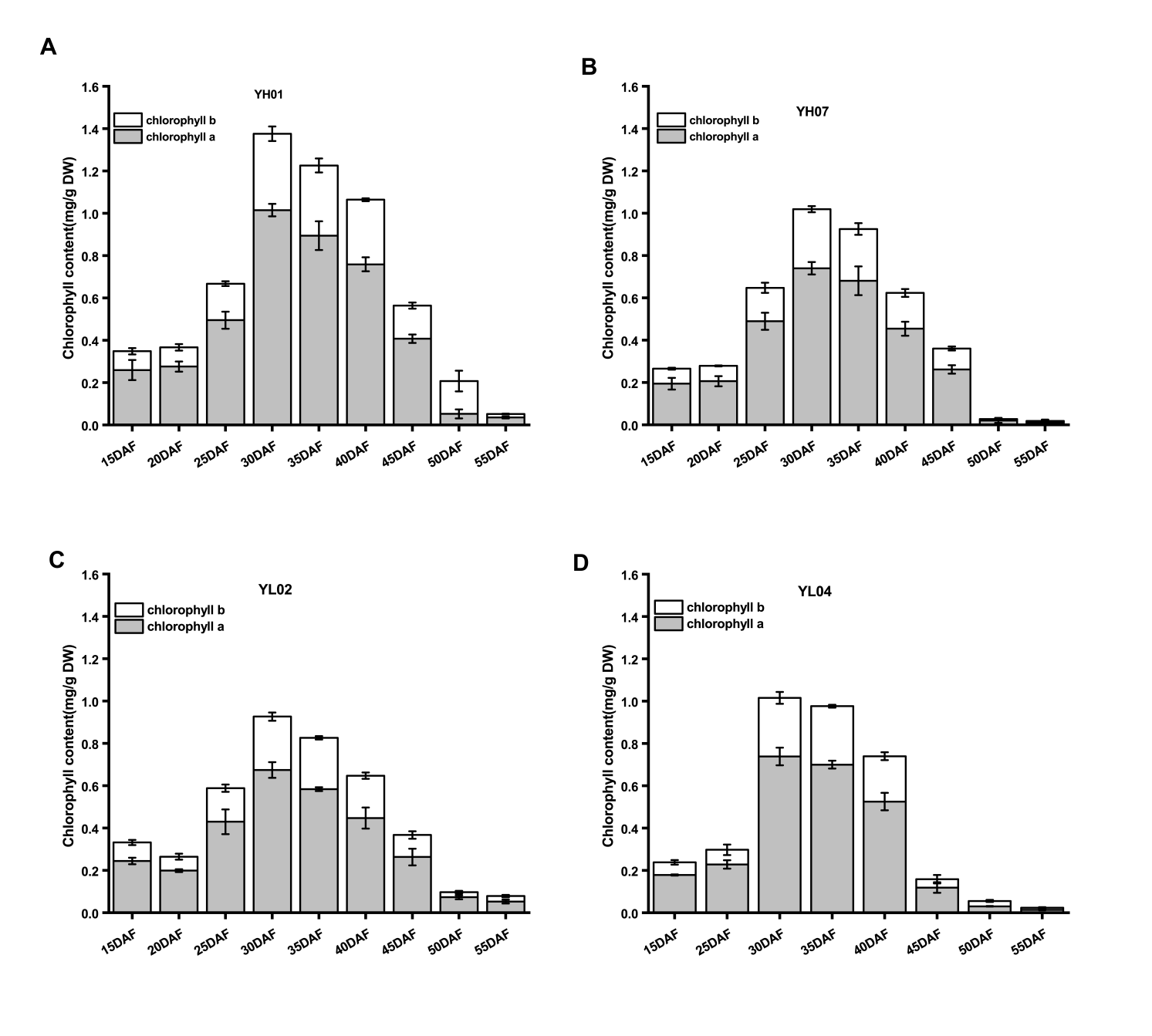
**Supplementary Figure S2.** Dynamic changes of chlorophyll content in rapeseed every 5 days starting from 15 days after flowering.

(A) Dynamic changes of chlorophyll content in YH01 rapeseed every 5 days starting from 15 days after flowering. (B) Dynamic changes of chlorophyll content in YH07 rapeseed every 5 days starting from 15 days after flowering. (C) Dynamic changes of chlorophyll content in YL02 rapeseed every 5 days starting from 15 days after flowering. (D) Dynamic changes of chlorophyll content in YL04 rapeseed every 5 days starting from 15 days after flowering. The results of each material are based on three biological and three technical replicates. The error bars indicate standard errors.


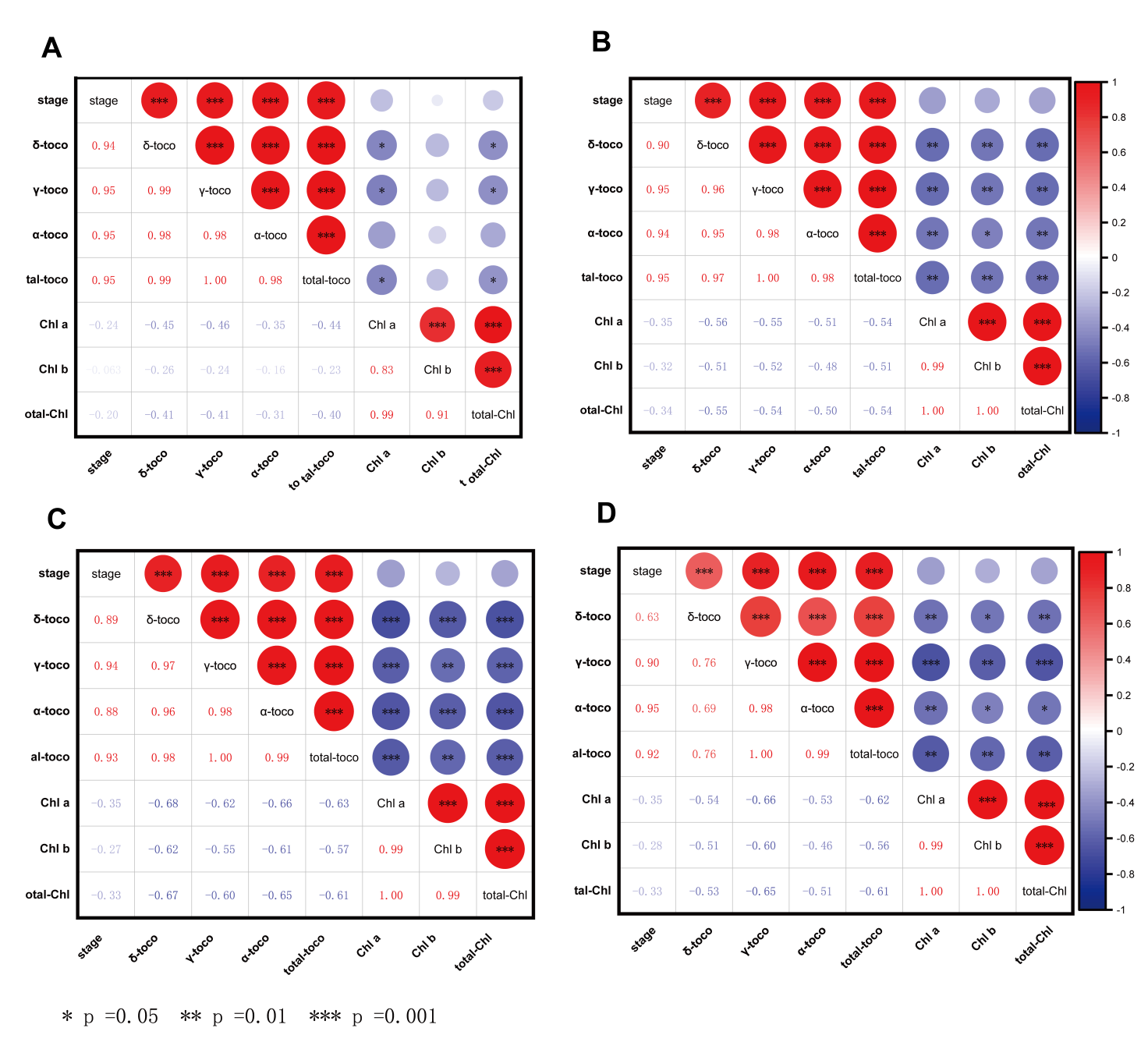


**Supplementary Figure S3.** Pearson correlations between tocochromanol compositions content and chlorophyll compositions content.

(A) Correlation analysis of tocopherol and chlorophyll components in YH01. (B) Correlation analysis of tocopherol and chlorophyll components in YH07. (C) Correlation analysis of tocopherol and chlorophyll components in YL02. (D) Correlation analysis of tocopherol and chlorophyll components in YL04. *, ** and *** indicate significant at the 0.05, 0.01 and 0.001 levels, respectively Red indicates positive correlation, blue indicates negative correlation, and colour depth indicates correlation


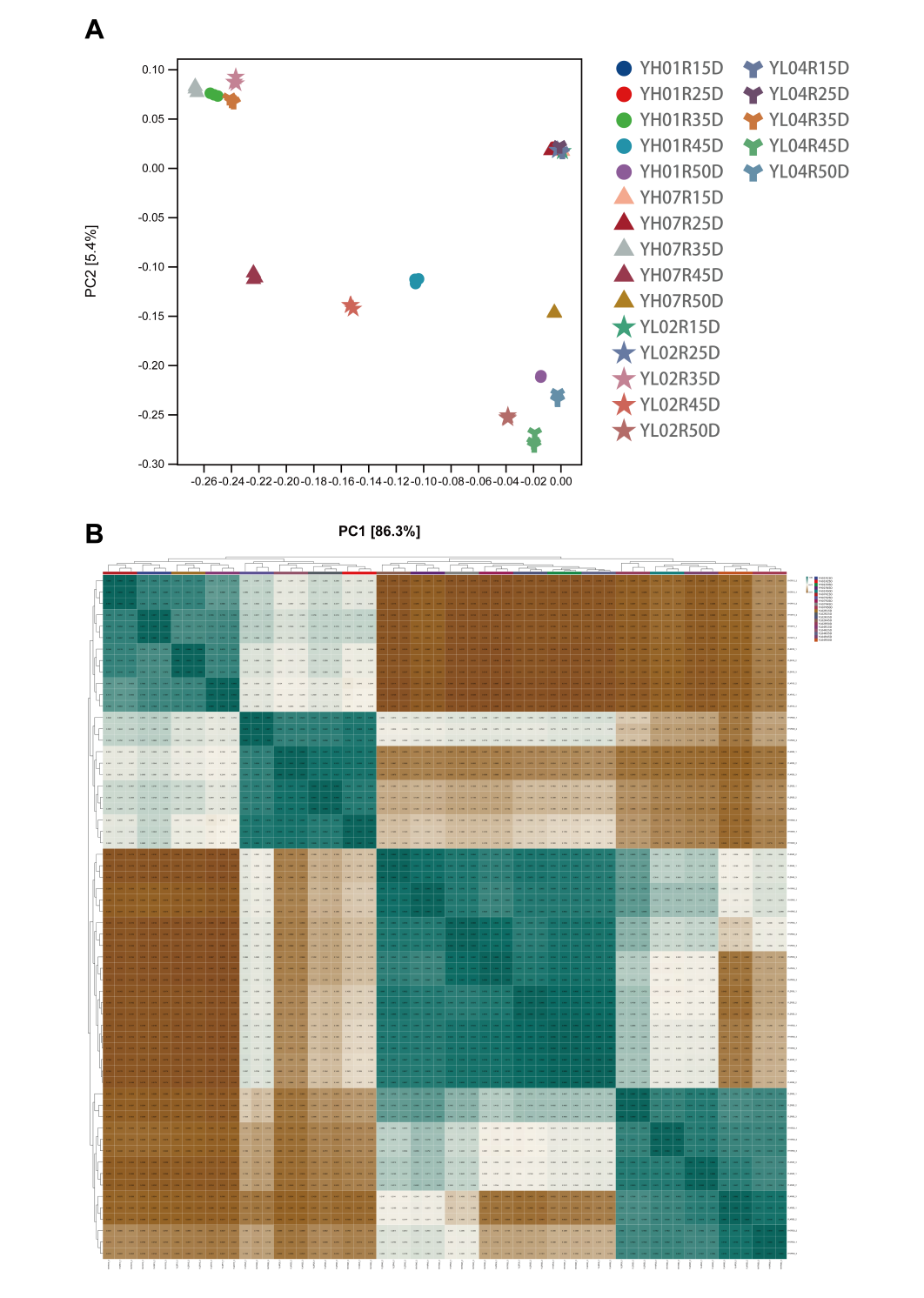


**Supplementary Figure S4.** Overview and reproducibility of the transcriptome.

(A)Principal component analysis (PCA) of twenty samples. (B)Heatmaps of Pearson correlation values of twenty samples.


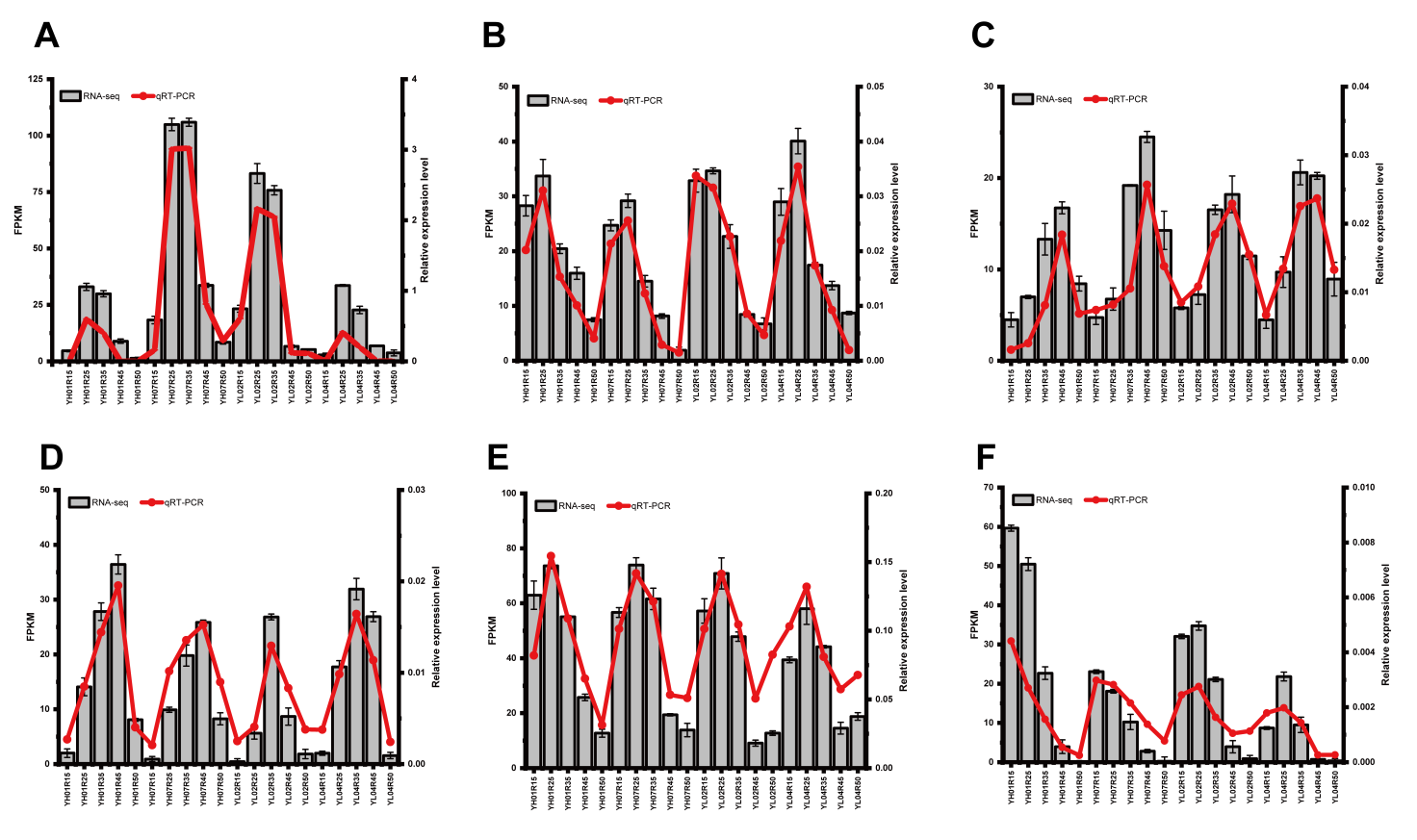


**Supplementary Figure S5.** Expression profiles of six genes by quantitative reverse transcription‐polymerase chain reaction (qRT‐PCR) and RNA‐seq.

(A-F) represent the expression levels of the homologs of BnA01g0030930, BnA01g0000070, BnA03g0154690, BnC03g0602410, BnA07g0285770 and BnA02g0061970, respectively. The left ordinates represent the relative expression levels determined by RNA‐seq, and right ordinates represents the relative expression levels determined by qRT‐PCR. The results of each gene are based on three biological and three technical replicates. The error bars indicate standard errors.
